# Cardiac infarction caused by PD-1 inhibitor during small cell neuroendocrine carcinoma of the ureter treatment: A case report

**DOI:** 10.3389/fonc.2023.1114397

**Published:** 2023-03-22

**Authors:** Xiaoying Li, Jing Wen, Hongtao Li, Yan Huang, Hongliang Zhou

**Affiliations:** Department of Oncology, the People’s Hospital of Yubei District of Chongqing City, Chongqing, China

**Keywords:** cardiac infarction, PD-1 inhibitor treatment, small cell neuroendocrine carcinoma the ureter, case report, literature review

## Abstract

Although small cell neuroendocrine carcinoma of the ureter (ureteral SCNEC) is rare, it always leads to a poor prognosis. Also, no treatment recommendation has been given for ureteral SCNEC, with only PD-1/PD-L1 inhibitors being used for its treatment. Here, we report a case of atypical symptoms of cardiac infarction caused by a PD-1 inhibitor used in the treatment of ureteral SCNEC and hope to address concerns regarding the possible cardiac toxicity caused by PD-1/PD-L1 inhibitors in ureteral SCNEC patients.

## Introduction

Small cell neuroendocrine carcinoma (SCNEC) is common in the lungs and constitutes a group of heterogeneous tumors originating from neuroendocrine cells of different organs. Primary SCNEC of the urinary system is rare, accounting for less than 0.5% of tumors in the urinary system (1), and is mainly found in the bladder and prostate. Additionally, small-cell neuroendocrine carcinoma of the ureter (ureteral SCNEC) is even extremely rare. Primary SCNEC of the ureter can progress rapidly with a poor prognosis, where most patients die within one year of diagnosis (2). The median survival period is 17 months, with 1- and 3-year survival rates being 51.9% and 30.3%, respectively (3). Moreover, the recurrence rate is as high as 60% (4). Currently, only 50 patients are reported with this tumor (5). Primary ureteral SCNEC is commonly seen in elderly men and is presented with gross hematuria and lumbar pain (4). However, with no recommendation for extensive or advanced disease, PD-1/PD-L1 inhibitor remains the treatment for ureteral SCNEC (6). Immune-related adverse events are a range of complications associated with the use of immune-checkpoint inhibitors what cantinas PD-1/PD-L1 inhibitors. High-grade immune-related adverse events are life-threatening and often cause a severe decline in performance status in such that patients do not qualify for any further anticancer treatments. Here, we report a case of cardiac infarction caused by the PD-1 inhibitor used for ureteral SCNEC treatment.

## Clinical data

**Figure 1:**
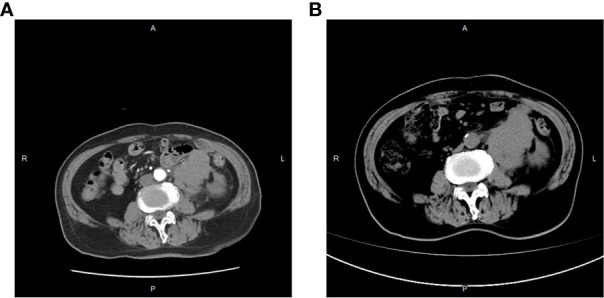
CT results. **(A)** CT result on April 25, 2022; **(B)** CT result on May 17, 2022.

**Figure 2:**
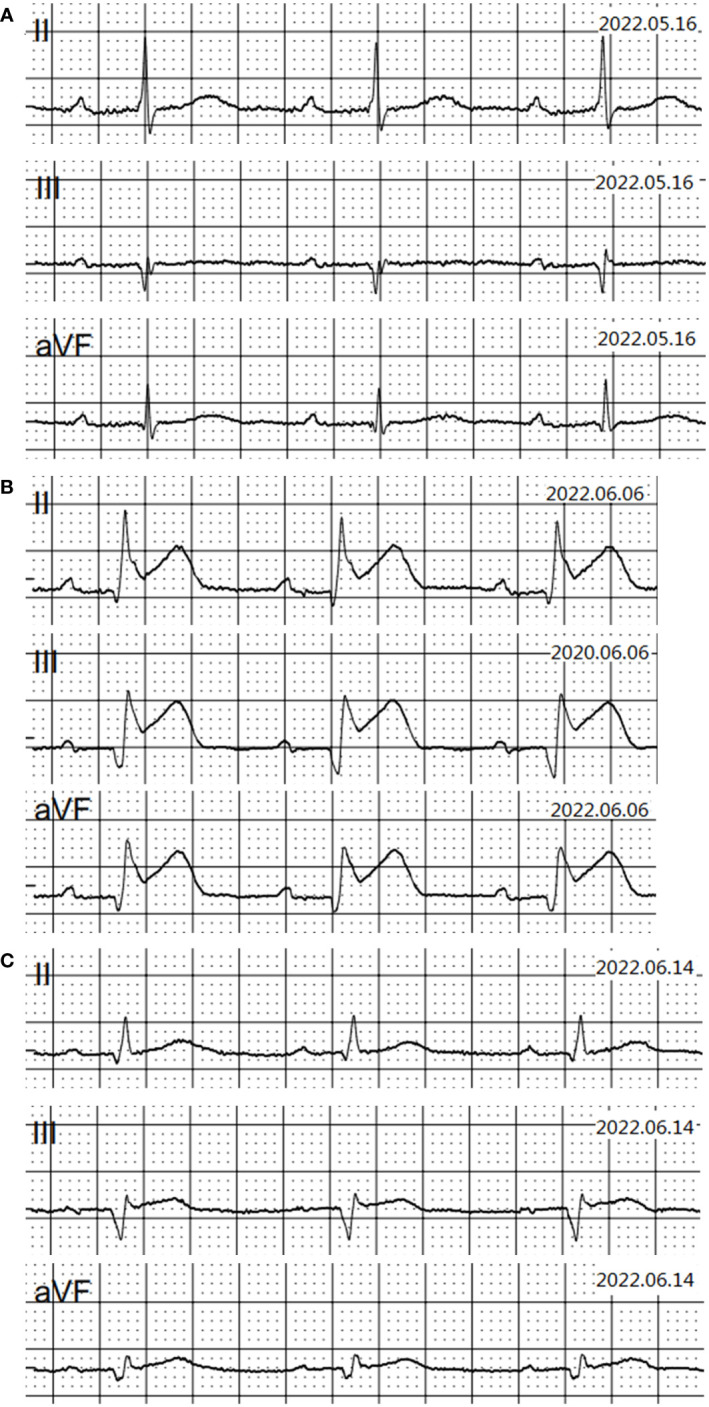
Electrocardiogram results. **(A)** electrocardiogram results on May 16, 2022; **(B)** electrocardiogram results on June 6, 2022; **(C)** electrocardiogram results on June 14, 2022.

**Figure 3:**
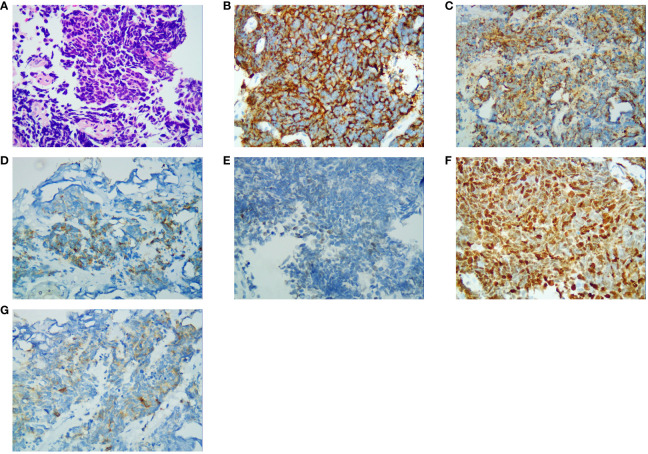
Immunohistochemical results (400×). **(A)** Hematoxylin and eosin-stained section result; **(B)** CD56 staining specific for neuroendocrine differentiation; **(C)** CK-L staining specific for neuroendocrine differentiation; **(D)** CK-pan staining specific for neuroendocrine differentiation; **(E)** GATA-3 staining specific for neuroendocrine differentiation; **(F)** Ki-67 staining specific for neuroendocrine differentiation; **(G)** Syn staining specific for neuroendocrine differentiation.

**Figure 4:**
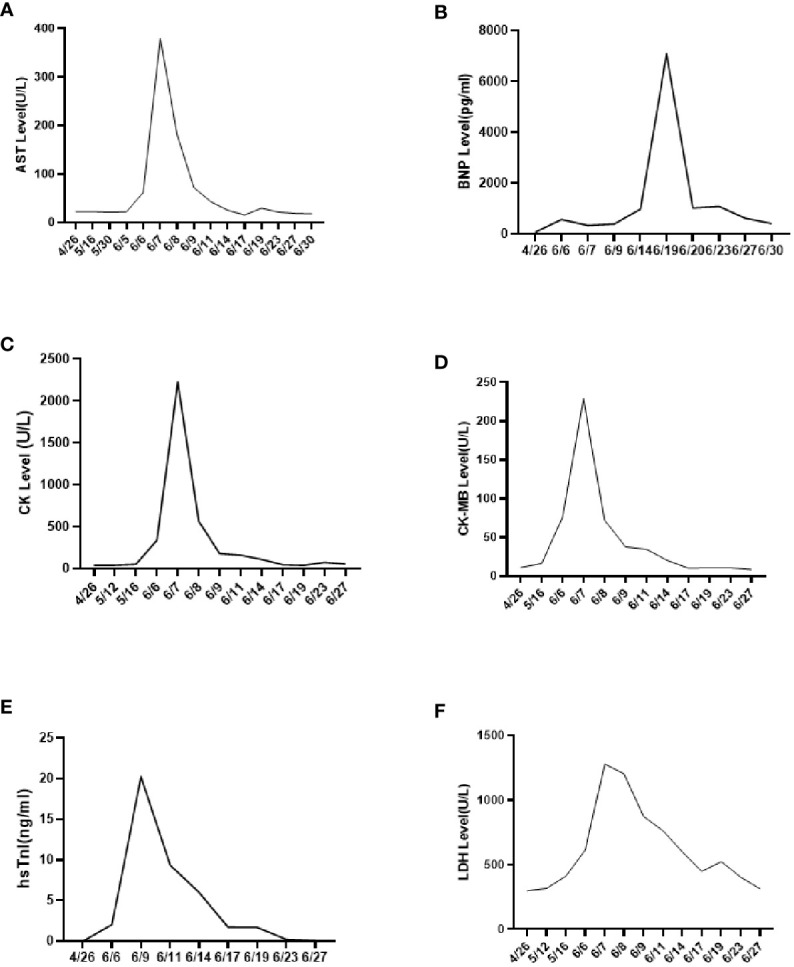
Cardiac enzyme profile examination results. **(A)** AST results; **(B)** BNP results; **(C)** CK results; **(D)** CK-MB results; **(E)** hsTnl results; **(F)** LDH results.

**Figure 5:**
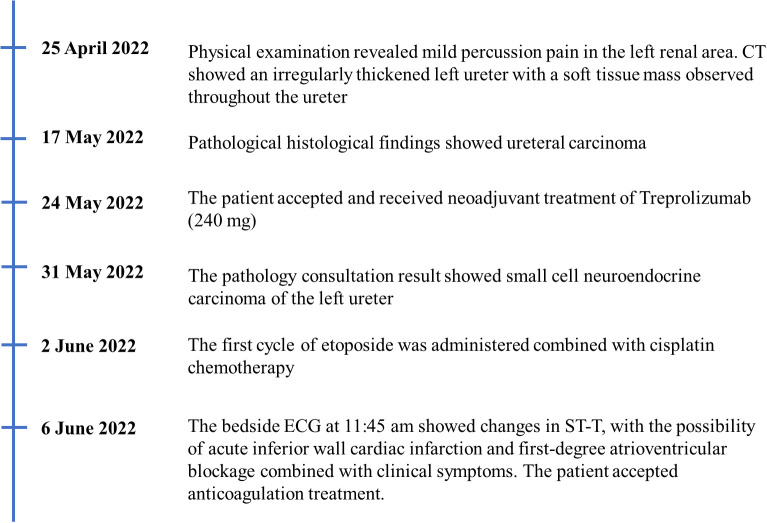
The timeline with relevant data from the episode of care.

On 25 April 2022, an 89-year-old male patient presented to our institution with right flank pain for more than 3 months. Physical examination revealed mild percussion pain in the left renal area. Contrast-enhanced CT showed an irregularly thickened left ureter with a soft tissue mass observed throughout the ureter (5.5cm×5.2cm, **Figure 1A**). Also, bilateral phonological changes were observed in both the lungs, along with emphysema. Arterial vascular imaging showed that both renal arteries originated from the abdominal aorta. Moreover, multiple calcified and non-calcified plaques were observed in both renal arteries, with varying degrees of luminal narrowing evident in the left renal artery. Calcified plaques were also seen in the abdominal aorta, superior and inferior mesenteric arteries, and common iliac artery, with mild local luminal narrowing. Ultrasound of arteries and veins of both lower limbs showed atherosclerosis with plaque formation. Cardiac color doppler ultrasound left heart function measurements, and tissue Doppler imaging (TDI) examinations revealed aortic sclerosis with aortic regurgitation (small amount), mitral and tricuspid regurgitation (small amount), and decompensation of left ventricular diastolic compliance. Routine ECG showed visible first-degree atrioventricular block and Q waves (**Figure 2A**). Cystoscopy results were as follows: pathological histological findings showed ureteral carcinoma, with a possibility of cT4N0M0 clinical stage. A laparotomy performed under general anesthesia showed intraoperative involvement of mesentery. Therefore, the operation was stopped, and no further surgical resection or biopsy of pathological tissue was performed. The clinical stage of cT4N0M1 was revised. On 17 May 2022, a plain CT scan of the whole abdomen was performed, which detected pneumoperitoneum along with irregular thickening of the wall throughout the left ureter and formation of a soft tissue mass (5.9 cm×5.3 cm), showing multiple surrounding lymph nodes (**Figure 1B**). The left renal pelvis showed heavy hydronephrosis with marked thinning on the left renal cortex and exudation around the left kidney, which was considered a tumorous lesion. A slightly dense nodule was observed in the right kidney with calcification of the prostate gland along with an increased density of the right inferior articular eminence in the lumbar 4. On 24 May 2022, the patient accepted and received neoadjuvant treatment of Treprolizumab (240 mg). Due to the patient’s history of coronary artery disease, we paid more attention to adverse reactions, especially cardiovascular issues. The immunohistochemical test revealed positive neuroendocrine markers (CD56, CK-L, CK-pan, GATA-3, Ki-67 and Syn), consistent with small cell neuroendocrine carcinoma (**Figure 3**). On 2 June 2022, the first cycle of etoposide was administered combined with cisplatin chemotherapy, which was as follows: etoposide 0.1 g D1-3 + nedaplatin 30 mg D1-3, Q3W. The patient was treated for hypotension using trimetazidine, which dilated the coronary arteries and improved myocardial blood supply, while atorvastatin calcium was given to regulate lipids along with other supportive and symptomatic treatments. On June 6, 2022, the patient experienced sudden nausea accompanied by heavy sweating. The bedside ECG at 11:45 am showed changes in ST-T, with the possibility of acute inferior wall cardiac infarction and first-degree atrioventricular blockage combined with clinical symptoms (**Figure 2B**). In contrast, bedside routine ECG at 12:34 am showed that lead III was off along with changes in ST-T, with ST-segment showing elevation in leads I, II, aVL, aVF at about 0.1 mv ~ 0.2 mv and ST segment showing depression in leads V1 ~ V6 at about 0.1 mv ~ 0.2 mv. Abnormal Q waves were seen in V1 V2 leads with the first-degree atrioventricular block (**Figure 2C**). Creatine kinase, lactate dehydrogenase, creatine kinase isoenzyme, aspartate aminotransferase, B-type brain natriuretic peptide precursor, and troponin I were found to be elevated at 338.03 (U/L), 615.43 (U/L), 75.53 (U/L), 61.58 (U/L), 569.00 (pg/mL), and 2.02 (ng/mL), respectively. After consultation, the patient was advised to undergo coronary artery stenting, which was refused by the family. The patient was then given anticoagulation treatment with nitroglycerin 10 mg + 0.9% NS, starting with 10 mL being pumped at 0.4 mL/h, which helped in expanding the coronary artery. Also, as recommended in the consultation, other supportive and symptomatic treatments were provided. The patient’s condition improved, and the cardiac enzyme profile and troponin showed a progressive decline to normal levels (**Figure 4**), and was discharged. A timeline with relevant data from the episode of care was showed in **Figure 5**.

## Discussion

The patient was admitted to the hospital due to soreness and pain on the left side of the waist. However, the CT scan showed an irregularly thickened wall throughout the left ureter along with soft tissue mass (5.5 cm×5.2 cm). These results, combined with the CT conducted on 17 May 2022, suggested the presence of a tumor (CT image).

Since the clinical manifestations and imaging results of ureteral SCNEC are not specific to other types of ureteral tumors, pathological detection of neuroendocrine markers, including the recommended Syn and CgA and CD56, remains an important method for diagnosing ureteral SCNEC (7). The immunohistochemical results of this patient indicated a poorly differentiated carcinoma with neuroendocrine differentiation; hence, it was diagnosed as SCNEC of the ureter.

Due to the extremely low incidence rate of ureteral SCNEC, its optimal treatment remains lacking. Kouba et al. believed that the pathological manifestations of primary urinary SCNEC were similar to those of small-cell lung cancer. Therefore, the clinical strategy of surgical resection and chemotherapy of small cell lung cancer can be considered as a reference for ureteral SCNEC (8). The surgical methods include radical nephroureterectomy and bladder cuff resection (9). However, the recurrence rate remains around 60% (10). Therefore, a comprehensive treatment is necessary for ureteral SCNEC. The cystoscopy and laparotomy showed that the ureteral carcinoma had involved the mesentery. Thus, due to the patient’s older age (89 years old) and physical condition, the patient’s family refused to undergo surgery. Platinum-based chemotherapy is reported to prolong the median survival of ureteral SCNEC patients (4). Qing et al. combined PD-L1 immune checkpoint inhibitors with radiotherapy to treat ureteral SCNEC and achieved good results (6). A previous study suggested that neoadjuvant chemotherapy may help in reducing the pathological staging of ureteral SCNEC (5). Based on the above evidence-based medical report, we tried to use the PD-1 inhibitor Treprizumab (240 mg) as a new adjuvant immunotherapy along with chemotherapy combining etoposide and cisplatin. After four days of starting chemotherapy (6 June 2022), the patient witnessed sudden nausea and sweating. The bedside ECG and myocardial zymogram examination revealed an acute myocardial infarction. Thus, the patient was given the symptomatic treatment of nitroglycerin and crown enlargement, which improved his condition (myocardial zymogram and electrocardiogram). PD-1/PD-L1 is the most widely used tumor immune checkpoint inhibitor that also significantly impacts the cardiovascular system (11). The presence of cancer and/or the use of PD-1/PD-L1 immune checkpoint inhibitors therapies can provoke changes in the organism, such as remodeling of immune cells, that affect the heart. Furthermore, specific oncometabolites, such D-2-hydroxyglutarate and succinate, can affect the heart tissue directly. Metabolic risk factors can cause cardiovascular disease as well as exacerbate tumor proliferation and cancer progression (12). Currently, the manifestations of cardiac toxicity caused by PD-1/PD-L1 immune checkpoint inhibitors include myocarditis, arrhythmia, conduction disease, myocardial infarction, pericardial disease, Takotsubo syndrome, non-inflammatory left ventricular dysfunction, etc. (13). A meta-analysis report showed that immune checkpoint inhibitors use was associated with an increased risk of 6 cardiovascular immune-related adverse events including myocarditis, pericardial diseases, heart failure, dyslipidemia, myocardial infarction, and cerebral arterial ischemia with higher risks for myocarditis and dyslipidemia (14). But the study based Chinese population reported the most common cardiotoxicity caused by immune checkpoint inhibitors was arrhythmia (9.3%) and 2.1% developed myocarditis in 5518 cancer patients who received at least one cycle of immune checkpoint inhibitors treatment (15). Therefore, clinicians need to focus on the cardiotoxicity of PD-1/PD-L1 immune checkpoint inhibitors, especially arrhythmia and myocarditis, before their clinical usage. Since this patient had a history of coronary heart disease, we closely observed the effects of the PD-1 inhibitor, Tereprimab. Even when the patient displayed nausea with sweating but no typical symptoms of myocardial infarction, we provided the supportive treatment of nitroglycerin in time to improve the bedside ECG and related laboratory examinations, significantly improving the patient’s symptoms. Although immunotherapy exhibits a good effect on tumors, elderly patients suffer from many basic diseases and generally poor physiques. Hence, more attention needs to be paid to adverse reactions, especially cardiovascular events, during immunotherapy. To avoid poor clinical outcomes, clinicians should fully consider the patient’s physical condition before immunotherapy, along with formulating an adverse reaction response plan and implementing it timely, if required. Since most immune-related adverse events resolve within weeks to months after the initiation of immunosuppressive therapy, one of the most important issues in clinical practice is the safety of resuming immune checkpoint blockade after the adverse event has resolved. Although recurrent adverse events are usually less severe than the initial events, a decision to restart treatment with immune checkpoint blockade is likely to depend on the severity of the prior event (16), the availability of alternative treatment options, and the overall status of the cancer. An absolute contraindication to restarting treatment with immune checkpoint blockade is life-threatening toxicity, particularly cardiac, pulmonary, or neurologic toxicity.

## Data availability statement

The raw data supporting the conclusions of this article will be made available by the authors, without undue reservation.

## Ethics statement

The studies involving human participants were reviewed and approved by the Ethics Committee of the People’s Hospital of Yubei District of Chongqing. The patients/participants provided their written informed consent to participate in this study. Written informed consent was obtained from the individual(s), and minor(s)’ legal guardian/next of kin, for the publication of any potentially identifiable images or data included in this article.

## Author contributions

XL was the leading principal investigator, designed the study, interpreted the data, wrote and revised the manuscript. JW and HL analyzed the data. YH and HZ collected the data. All authors contributed to the article and approved the submitted version.

## References

[1] Acosta AM Kajdacsy-Balla A. Primary neuroendocrine tumors of the ureter: A short review. Arch Pathol Lab Med (2016) 140(7):714–7. doi: 10.5858/arpa.2015-0106-RS 27362572

[2] Hensley PJ Bhalodi AA Gupta S. Primary upper urinary tract small cell carcinoma: A case series and literature review. J Endourol Case Rep (2017) 3(1):165–8. doi: 10.1089/cren.2017.0103 PMC5699520 29177194

[3] Zhong W Lin R Zhang L Jin C Li X He Q. Clinicopathologic characteristics, therapy and outcomes of patients with primary ureteral small cell carcinoma: a case series and systematic review of the literature. Onco Targets Ther (2017) 10:4105–11. doi: 10.2147/OTT.S138769 PMC5566501 28860819

[4] Ouzzane A Ghoneim TP Udo K Verhasselt-Crinquette M Puech P Betrouni N. Small cell carcinoma of the upper urinary tract (UUT-SCC): report of a rare entity and systematic review of the literature. Cancer Treat Rev (2011) 37(5):366–72. doi: 10.1016/j.ctrv.2010.12.005 21257269

[5] Farci F Manassero F Baldesi R Bartolucci A Boldrini L Selli C. Primary small cell carcinoma of the ureter: Case report and review of the literature. Med (Baltimore) (2018) 97(24):e11113. doi: 10.1097/MD.0000000000011113 PMC6023684 29901633

[6] Qing D Peng L Cen F Huang X Wei Q Lu H. Hyperprogression after immunotherapy for primary small cell neuroendocrine carcinoma of the ureter: A case report. Front Oncol (2021) 11:696422. doi: 10.3389/fonc.2021.696422 34485132 PMC8416087

[7] Guo AT Chen W Wei LX. Clinical and pathologic characteristics of small cell neuroendocrine carcinoma of urinary tract. Zhonghua Bing Li Xue Za Zhi (2012) 41(11):747–51. doi: 10.3760/cma.j.issn.0529-5807.2012.11.008 23302335

[8] Kouba EJ Cheng L. Understanding the genetic landscape of small cell carcinoma of the urinary bladder and implications for diagnosis, prognosis, and treatment: A review. JAMA Oncol (2017) 3(11):1570–8. doi: 10.1001/jamaoncol.2016.7013 28334324

[9] Maruyama Y Araki M Wada K Yoshinaga K Mitsui Y Sadahira T. Long-term ureteroscopic management of upper tract urothelial carcinoma: 28-year single-centre experience. Jpn J Clin Oncol (2021) 51(1):130–7. doi: 10.1093/jjco/hyaa132 32715306

[10] Majhail NS Elson P Bukowski RM. Therapy and outcome of small cell carcinoma of the kidney: report of two cases and a systematic review of the literature. Cancer (2003) 97(6):1436–41. doi: 10.1002/cncr.11199 12627507

[11] Khunger A Battel L Wadhawan A More A Kapoor A Agrawal N. New insights into mechanisms of immune checkpoint inhibitor-induced cardiovascular toxicity. Curr Oncol Rep (2020) 22(7):65. doi: 10.1007/s11912-020-00925-8 32514647

[12] Karlstaedt A Moslehi J de Boer RA. Cardio-onco-metabolism: metabolic remodelling in cardiovascular disease and cancer. Nat Rev Cardiol (2022) 19(6):414–25. doi: 10.1038/s41569-022-00698-6 PMC10112835 35440740

[13] Shalata W Abu-Salman A Steckbeck R Mathew Jacob B Massalha I Yakobson A. Cardiac Toxicity Associated with Immune Checkpoint Inhibitors: A Systematic Review. Cancers (Basel) (2021) 13(20):5218. 34680365 10.3390/cancers13205218 PMC8534225

[14] Dolladille C Akroun J Morice PM Dompmartin A Ezine E Sassier M. Cardiovascular immunotoxicities associated with immune checkpoint inhibitors: a safety meta-analysis. Eur Heart J (2021) 42(48):4964–77. doi: 10.1093/eurheartj/ehab618 34529770

[15] Li C Bhatti SA Ying J. Immune checkpoint inhibitors-associated cardiotoxicity. Cancers (Basel) (2022) 14(5):1145. doi: 10.3390/cancers14051145 35267453 PMC8909315

[16] Postow MA Sidlow R Hellmann MD. Immune-related adverse events associated with immune checkpoint blockade. N Engl J Med (2018) 378(2):158–68. doi: 10.1056/NEJMra1703481 29320654

