# Corrigendum: Cardiac infarction caused by PD-1 inhibitor during small cell neuroendocrine carcinoma of the ureter treatment: a case report

**DOI:** 10.3389/fonc.2024.1438794

**Published:** 2024-07-05

**Authors:** Xiaoying Li, Jing Wen, Hongtao Li, Yan Huang, Hongliang Zhou

**Affiliations:** Department of Oncology, the People’s Hospital of Yubei District of Chongqing City, Chongqing, China

**Keywords:** cardiac infarction, PD-1 inhibitor treatment, small cell neuroendocrine carcinoma the ureter, case report, literature review

In the published article, there was an error in **Figure 3G** as published. We used the wrong image in **Figure 3G** which is duplicated with **Figure 3F**. The corrected **Figure 3** and its caption “Immunohistochemical results (400×). (A) Hematoxylin and eosin-stained section result; (B) CD56 staining specific for neuroendocrine differentiation; (C) CK-L staining specific for neuroendocrine differentiation; (D) CK-pan staining specific for neuroendocrine differentiation; (E) GATA-3 staining specific for neuroendocrine differentiation; (F) Ki-67 staining specific for neuroendocrine differentiation; (G) Syn staining specific for neuroendocrine differentiation” appear below.

**Figure 3:**
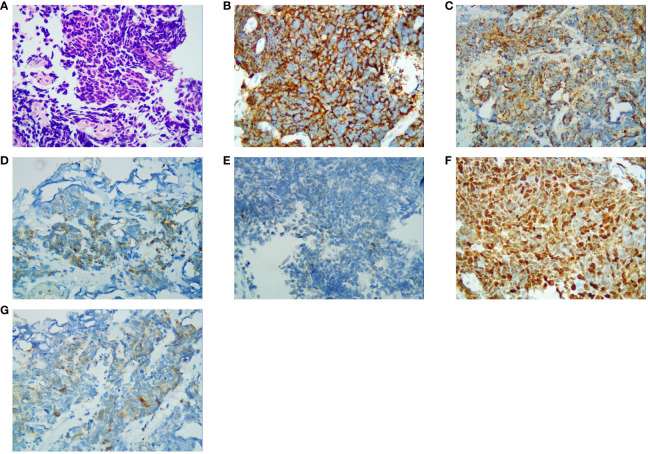
Immunohistochemical results (400×). **(A)** Hematoxylin and eosin-stained section result; **(B)** CD56 staining specific for neuroendocrine differentiation; **(C)** CK-L staining specific for neuroendocrine differentiation; **(D)** CK-pan staining specific for neuroendocrine differentiation; **(E)** GATA-3 staining specific for neuroendocrine differentiation; **(F)** Ki-67 staining specific for neuroendocrine differentiation; **(G)** Syn staining specific for neuroendocrine differentiation.

The authors apologize for this error and state that this does not change the scientific conclusions of the article in any way. The original article has been updated.

